# Transitions of CDR-L3 Loop Canonical Cluster Conformations on the Micro-to-Millisecond Timescale

**DOI:** 10.3389/fimmu.2019.02652

**Published:** 2019-11-19

**Authors:** Monica L. Fernández-Quintero, Barbara A. Math, Johannes R. Loeffler, Klaus R. Liedl

**Affiliations:** Center for Molecular Biosciences Innsbruck (CMBI), Institute of General, Inorganic and Theoretical Chemistry, University of Innsbruck, Innsbruck, Austria

**Keywords:** canonical structures, CDR-L3 loop, molecular dynamics simulations, markov-state models, conformational ensemble, antibody structure design

## Abstract

Sequence and structural diversity of antibodies are concentrated on six hypervariable loops, also known as the complementarity determining regions (CDRs). Five of six antibody CDR loops presumably adopt a so-called canonical structure out of a limited number of conformations. However, here we show for four antibody CDR-L3 loops differing in length and sequence, that each loop undergoes conformational transitions between different canonical structures. By extensive sampling in combination with Markov-state models we reconstruct the kinetics and probabilities of the transitions between canonical structures. Additionally, for these four CDR-L3 loops, we identify all relevant conformations in solution. Thereby we extend the model of static canonical structures to a dynamic conformational ensemble as a new paradigm in the field of antibody structure design.

## Introduction

Antibodies have become key players as therapeutic agents and therefore the understanding of the antigen-binding process is crucial (1, 2). The antibody binding site consists of six hypervariable loops, each three on the variable domains of the heavy (V_H_) and the light chain (V_L_) that shape the antigen binding site, the paratope (2–5). Five of the six antibody CDR loops can adopt a limited number of main-chain conformations known as canonical structures, except of the CDR-H3 loop (6–8). The CDR-H3 loop, due to its high diversity in length, sequence and structure and its ability to adopt various different conformations during the V(D)J recombination and somatic hyper-mutation, remains challenging to predict accurately (9–13). Together with the CDR-H3 loop the CDR-L3 loop is situated in the center of the paratope and contributes to antigen recognition (14). The CDR-L3 loop is similarly diverse, however without the contribution of a D gene the degree of variability is less (15). The CDR-L3 loop reveals a diversity of length and sequence composition due to the recombination of two gene segments V_L_ and J_L_. The V_L_ segment codes for the residues 1–95, including the first two CDR loops, while the CDR-L3 loop is encoded by the end of the V_L_ and the beginning of the J_L_ segment (16). The most prominent CDR-L3 loop length consists of nine residues and can adopt six possible canonical clusters. The rarest CDR-L3 loops contain 7, 12 and 13 residues and have only one canonical cluster (17, 18). However, even due to the increase in the number of crystal structures and consequentially also in canonical structures, the relative populations of the canonical clusters are expected to stay the same (15). There are two types of light chains, kappa and lambda. The genes encoding the two light chains are located on separate chromosomes. Kappa gene segments are encoded on chromosome 2 (52 V genes and 5 J genes) whereas lambda gene segments are encoded on chromosome 22 (30 V genes and 7 J genes) (16, 19–22). Depending on the type of light chain, antibodies reveal differences in conformational flexibility, half-life, and specificity (23). Various studies focused on classifying antibody structures and correlated it with their locus and sequence to improve antibody structure prediction and design (24–27). Additionally there exist several numbering systems for antibodies that are similar in the framework region, but differ around the CDRs (6, 28–30). The PyIgClassify database classifies conformational clusters by determining the CDR sequences and lengths using the IMGT nomenclature (28) and calculating the dihedral angles ω, φ, and ψ of the residues in each CDR (27). We analyzed the conformational diversity of the CDR-L3 loop to identify transition probabilities and timescales between canonical CDR-L3 loop conformations of same length and to characterize the CDR-L3 loop in solution. We focused on the CDR-L3 loop, because it reveals a diversity in sequence and structure comparable to the CDR-H3 loop.

## Methods

A previously published method characterizing the CDR-H3 loop ensemble in solution (31, 32) was used to investigate the conformational diversity of CDR-L3 loops. Experimental structure information was available for all considered antibody fragments (Fvs). The starting structures for simulations were prepared in MOE (Molecular Operating Environment, Chemical Computing Group, version 2018.01) using the Protonate3D tool (33, 34). To neutralize the charges we used the uniform background charge (35–37). Using the tleap tool of the AmberTools16 (35, 36) package, the crystal structures were soaked with cubic water boxes of TIP3P water molecules with a minimum wall distance of 10 Å to the protein (38). For all crystal structures parameters of the AMBER force field 14SB were used (39). The antibody fragments were carefully equilibrated using a multistep equilibration protocol (40).

### Metadynamics Simulations

To enhance the sampling of the conformational space well-tempered metadynamics (41–43) simulations were performed in GROMACS (44, 45) with the PLUMED 2 implementation (46). As collective variables, we used a linear combination of sine and cosine of the ψ torsion angles of the CDR-H3 and CDR-L3 loop calculated with functions MATHEVAL and COMBINE implemented in PLUMED 2 (46). As discussed previously, the ψ torsion angle captures conformational transitions comprehensively (38, 39). The decision to include the CDR-L3 and CDR-H3 loop ψ torsion angles is based on the structural correlation of the CDR-L3 and CDR-H3 loop and the observed improved sampling efficiency (47). The simulations were performed at 300 K in an NpT ensemble. We used a Gaussian height of 10.0 kcal/mol. Gaussian deposition occurred every 1,000 steps and a biasfactor of 10 was used. 1 μs metadynamics simulations were performed for each available antibody fragment crystal structure. We applied an average linkage hierarchical clustering algorithm with a distance cut-off criterion of 1.2 Å on the resulting trajectories in cpptraj (36, 48) to obtain a large number of clusters.

The cluster representatives for the antibody fragments were equilibrated and simulated for 100 ns using the AMBER16 (35) simulation package.

### Molecular Dynamics Simulations

Molecular dynamics simulations were performed in an NpT ensemble using pmemd.cuda (49). Bonds involving hydrogen atoms were restrained by applying the SHAKE algorithm (50), allowing a time step of 2.0 fs. Atmospheric pressure of the system was preserved by weak coupling to an external bath using the Berendsen algorithm (51). The Langevin thermostat (52) was used to maintain the temperature during simulations at 300 K.

An in-house python hierarchical clustering script using pytraj (36, 53, 54) was used to directly calculate the transitions between the CDR-L3 loop cluster representatives within one simulation. To obtain a representative ensemble in solution and to account for different inherent CDR-L3 loop flexibilities the distance cut-off was chosen for each antibody individually. This clustering is only used to visualize the frequency of transitions, but it is not used for any further analyses. Within these resulting clusters most of the canonical conformation median crystal structures are found. Depending on the CDR-L3 loop length a different number of canonical clusters are available and the median crystal structure information for each loop length was extracted from the PyIgClassify database (27).

Separately, a time-lagged independent component analysis (tICA) was performed using the python library PyEMMA 2 employing a lag time of 10 ns (55). Additionally, PyEMMA 2 was chosen to calculate a Markov-state model (56) to reconstruct the thermodynamics and kinetics, using the k-means clustering algorithm (57) to define microstates and the PCCA+ clustering algorithm (58) to coarse grain the microstates to macrostates. The sampling efficiency and the reliability of the Markov-state model (e.g., defining optimal feature mappings) can be evaluated with the Chapman-Kolmogorov test (59, 60), by using the variational approach for Markov processes (61) and by taking into account the fraction of states used, as the network states must be fully connected to calculate probabilities of transitions and the relative equilibrium probabilities. To build the Markov-state model we used the backbone torsions of the CDR-L3 loop, defined 150 microstates using the k-means clustering algorithm and applied a lag time of 10 ns.

## Results

**Figure 1:**
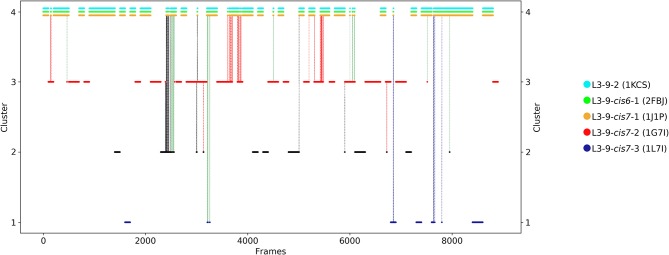
Conformational transitions of the CDR-L3 loop within the obtained 8.9 μs trajectories. This plot shows the number of clusters as a function of frames. The vertical lines in this plot show transitions between the clusters during each 100 ns of molecular dynamics simulations and are colored according to the cluster the simulation was started from. The canonical cluster medians can be observed within the CDR-L3 loop ensemble in solution. Within simulated cluster 4, which is the highest populated cluster, three canonical structure medians can be identified, while in simulated cluster 3 only one canonical structure can be found.

**Figure 2:**
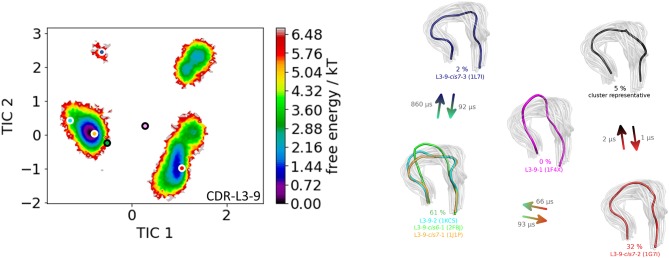
On the left the tICA plot of the 8.9 μs molecular dynamics trajectories with the projected six canonical structure medians for the CDR-L3 loop with a loop length of nine residues is shown. On the right the Markov-state model of the CDR-L3 loop is illustrated, displaying the probabilities and timescales of conformational transitions. The canonical structure medians are color-coded according to the tICA plot with a representative CDR-L3 loop ensemble in the background. An additional potentially important macrostate representative was identified and is colored gray.

The first antibody variable fragment (Fv) studied is the house dust mite allergen binding antibody. Der p 1 and Der f 1 are potent allergens, produced by house dust mites, and cause allergic sensitization and asthma. The PDB structures 3RVW (crystallized with antigen) and 3RVT (crystallized without antigen) were simulated without the antigen present (62). The CDR-L3 loop length of this house dust mite allergen binding antibody is nine residues. The crystal structures of the 3RVT and 3RVW were originally assigned to the L3-9-cis7-1 cluster containing 1,554 crystal structures, which is the highest populated canonical cluster with the CDR-L3 loop length of nine residues. The PDB accession code of this canonical cluster median is 1J1P, which is colored-coded orange in all following pictures. Besides the characterization of the CDR-H3 loop as conformational ensemble this approach allows to describe the CDR-L3 loop ensemble in solution. As described in the methods section the resulting 89 cluster representatives of the metadynamics simulations were simulated for each 100 ns molecular dynamics simulations. The resulting 8.9 μs trajectories were clustered using a hierarchical clustering algorithm with a distance cut-off of 2.4 Å. Figure 1 shows the conformational transitions observed within the 89 molecular dynamics simulations of 100 ns each. Cluster 4 is the highest populated cluster in which we found three of the six available canonical structure medians (L3-9-2, L3-9-cis6-1, and L3-9-cis7-1) of the CDR-L3 loop with residue length 9. The canonical structure median identified within cluster 3 belongs to the canonical cluster L3-9-cis7-2. The canonical cluster median PDB 1L7I of the L3-9-cis7-3 was found in the very low populated first cluster. The PDB 1F4X belongs to the L3-9-1 canonical structure and is not found in the simulations. Figure 1 shows various conformational transitions between the four clusters which means that we observe conformational transitions between the canonical structures of the CDR-L3 loop. To identify transition kinetics of the CDR-L3 loop ensemble in solution we calculated a Markov-state model based on a tICA by using the backbone torsions of the CDR-L3 loop (Figure 2). Figure 2 clearly confirms the results of the cluster analysis in Figure 1. Combined with a fully connected Markov-state model we identified four macrostates, in which five of the six canonical structures are present. Surprisingly, we even find three canonical structures, including the assigned median canonical structure of the L3-9-cis7-1 canonical cluster 1J1P in the same global minimum in solution. The transitions between the two highest populated macrostates occur in the low microsecond timescale, while the conformational transitions to the least probable macrostate, in which the canonical cluster median of the L3-9-1 1L7I is sampled, occur in the micro-to-millisecond timescale. The canonical cluster median 1F4X, colored in magenta, was not observed.

**Figure 3:**
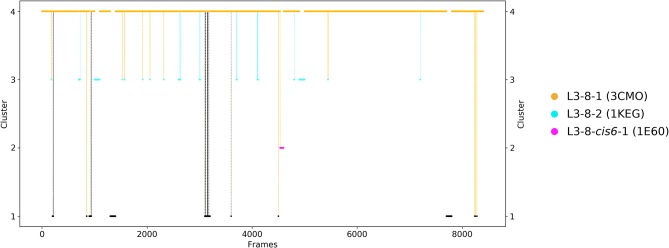
Conformational transitions of the CDR-L3 loop within the obtained 8.6 μs trajectories. This plot shows the number of clusters as a function of frames. The vertical lines in this plot show transitions between the clusters during each 100 ns of molecular dynamics simulations and are colored according to the cluster the simulation was started from. The canonical cluster medians can be observed within the CDR-L3 loop ensemble in solution. Within simulated cluster 4, which is the highest populated cluster, the predicted canonical structure median with the PDB code 3CMO is present. The canonical structure with the PDB code 1KEG can be found in simulated cluster 3. The third canonical structure median with the PDB code 1E6O is present in the lowest populated cluster 2.

**Figure 4:**
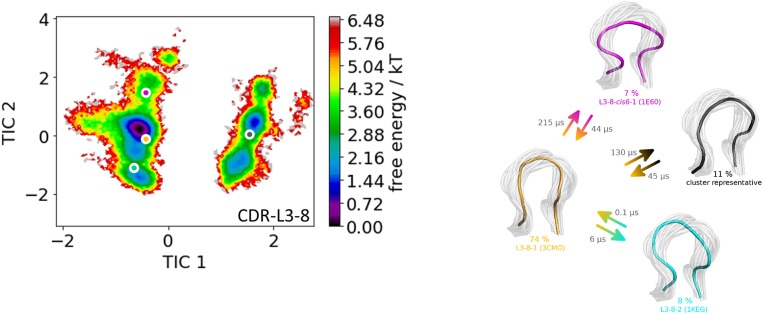
On the left the tICA plot of the 8.6 μs molecular dynamics trajectories with the projected three canonical structure medians for the CDR-L3 loop with a loop length of eight residues is shown. An additional macrostate representative of the CDR-L3 loop is projected in gray. On the right the Markov-state model of the CDR-L3 loop is illustrated, displaying the probabilities and timescales of conformational transitions. The canonical structure medians are color-coded according to the tICA plot with a representative CDR-L3 loop ensemble in the background.

As a second antibody Fv fragment to characterize the CDR-L3 loop ensemble in solution, the antibody binding to lymphocyte function—associated antigen-1 integrin (LFA-1 integrin) was analyzed. LFA-1 integrin plays a vital role in adhesive interactions with both endothelial cells and antigen-presenting cells (63). Again, the two crystal structures 3HI6 (crystallized with antigen) and 3HI5 (crystallized without antigen) were simulated without antigen present. The CDR-L3 loop contains eight residues and the crystal structures were assigned to the canonical CDR-L3 loop cluster L3-8-1. Figure 3 shows the clustering transitions of the obtained 8.6 μs molecular dynamics simulations with a distance cut-off of 1.4 Å. Within the highest populated cluster 4 the assigned canonical cluster median crystal structure 3CMO is present. The canonical cluster median of the cluster L3-8-2 (PDB 1KEG) is present within cluster 3. Within the least probable cluster 2 the rarest occurring canonical cluster for this loop length L3-8-cis6-1 consisting of only four crystal structures can be found. Besides the sampling of all available canonical conformations of the CDR-L3 loop with eight residues we also observe in Figure 3 another possible CDR-L3 loop conformation in solution. To identify the kinetic and thermodynamic role of the sampled conformations again a tICA in combination with a Markov-state model was performed (Figure 4). Figure 4 shows the probabilities and transition kinetics of the CDR-L3 loop ensemble in solution. The three available canonical median structures of the CDR-L3 loop are color-coded according to the clustering in Figure 3. Again, the assigned canonical cluster median structure 3CMO is present in the highest populated macrostate of the free energy landscape, which is in line with the hierarchical clustering in Figure 3. The transitions between the canonical cluster medians 3CMO and 1KEG occur in the nano-to-microsecond timescale, while the conformational transitions to the least probable macrostate show high microsecond timescales, in which the third canonical structure 1E6O was found.

**Figure 5:**
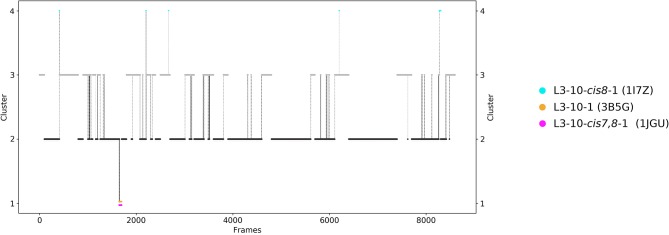
Conformational transitions of the CDR-L3 loop within the obtained 8.5 μs trajectories. This plot shows the number of clusters as a function of frames. The vertical lines in this plot show transitions between the clusters during each 100 ns of molecular dynamics simulations and are colored according to the cluster the simulation was started from. The canonical cluster medians can be observed within the CDR-L3 loop ensemble in solution. Within simulated cluster 4, which is the least populated cluster, the predicted canonical structure medians with the PDB code 1JGU and 3B5G are present. The canonical structure with the PDB code 1I7Z can be found in simulated cluster 1.

**Figure 6:**
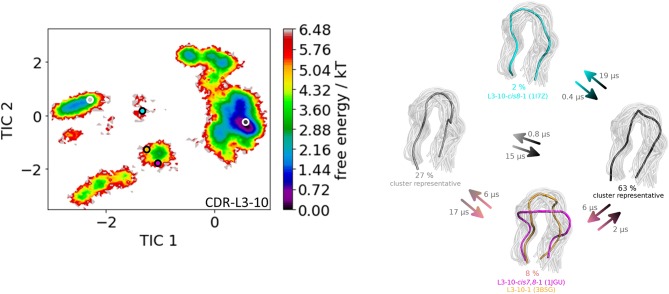
On the left the tICA plot of the 8.5 μs molecular dynamics trajectories with the projected three canonical structure medians for the CDR-L3 loop with a loop length of 10 residues is shown. Additional macrostate representatives of the CDR-L3 loop are projected in gray. On the right the Markov-state model of the CDR-L3 loop is illustrated, displaying the probabilities and timescales of conformational transitions. The canonical structure medians are color-coded according to the tICA plot with a representative CDR-L3 loop ensemble in the background.

The third analyzed antibody Fv fragment is binding interleukin-13 (IL-13), which is a member of the growth-hormone-like cytokine family and plays a central role in the development of asthma (64, 65). Again, two crystal structures (3G6D and 3G6A) were available and simulated without antigen present. The CDR-L3 loop length of this IL-13 binding antibody is 10 residues. For this IL-13 binding antibody, because of its length, sequence composition and type of light chain (lambda) no canonical cluster could be assigned by sequence comparison. We compared the resulting CDR-L3 loop ensemble in solution to the available three canonical cluster medians of the same length. Figure 5 shows the results of the hierarchical clustering of 8.5 μs molecular dynamics trajectories of the CDR-L3 loop ensemble using a distance cut-off of 1.6 Å. Within the low populated cluster 4 we find canonical cluster median crystal structures 1JGU and 3B5G of the canonical clusters L3-10-cis7,8-1 and the L3-10-1, respectively. Within the least populated cluster 1 we were also able to identify the third canonical cluster median 1I7Z of the canonical cluster L3-10-cis8-1. Besides sampling transitions between the canonical clusters, we observed the highly populated clusters 2 and 3 showing various conformational transitions. To retain the kinetics and state probabilities a Markov-state model was performed to identify the dominant CDR-L3 loop solution structures. Figure 6 displays the free energy surface with the projected canonical cluster representatives, color-coded according to Figure 5. Besides the local shallow side minima, in which the canonical cluster median structures are lying, Figure 5 shows a broad free energy surface indicating the existence of other more probable and dominant CDR-L3 loop conformations in solution. The transitions between the four macrostates of this antibody occur in the nano-to-microsecond timescale, in which we again observed transitions between canonical structures.

**Figure 7:**
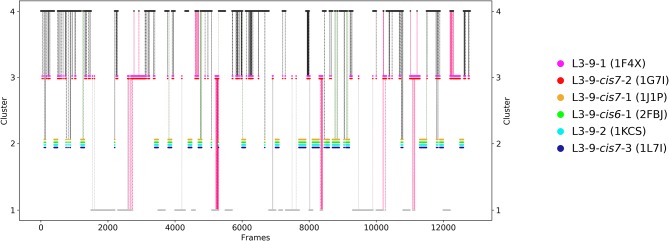
Conformational transitions of the CDR-L3 loop within the obtained 12.7 μs trajectories. This plot shows the number of clusters as a function of frames. The vertical lines in this plot show transitions between the clusters during each 100 ns of molecular dynamics simulations and are colored according to the cluster the simulation was started from. The canonical cluster medians can be observed within the CDR-L3 loop ensemble in solution. Within cluster 3 two canonical structure were present, and within cluster 2 even four canonical clusters were sampled.

**Figure 8:**
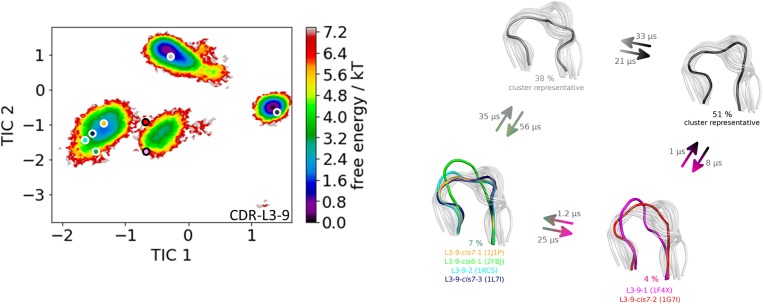
On the left the tICA plot of the 12.7 μs molecular dynamics trajectories with the projected three canonical structure medians for the CDR-L3 loop with a loop length of nine residues is shown. Two additional macrostate representatives of the CDR-L3 loop are projected in different shades of gray. On the right the Markov-state model of the CDR-L3 loop is illustrated, displaying the probabilities and timescales of conformational transitions. The canonical structure medians are color-coded according to the tICA plot with a representative CDR-L3 loop ensemble in the background.

The last antibody Fv fragment investigated is the anti-hemagglutinin binding influenza antibody (66). Three crystal structures were available (PDB codes 1HIM, 1HIN, 1HIL) and simulated without antigen present. This anti-hemagglutinin binding antibody has a CDR-L3 loop length of nine residues and the available crystal structures were assigned to the highest populated canonical cluster L3-9-cis7-1 with the median crystal structure 1J1P. The obtained 12.7 μs molecular dynamics trajectories were clustered with a distance cut-off of 1.1 Å and the conformational transitions are shown in Figure 7. Within cluster 3 the median crystal structures of the canonical clusters L3-9-cis7-2 (cluster median 1G7I) and the L3-9-1 (cluster median 1F4X) were sampled. The other four available canonical cluster medians for the CDR-L3 loop length of nine residues were found in cluster 2. According to the hierarchical clustering the highest populated clusters are cluster 4 and cluster 1. Figure 8 displays the Markov-state model of the CDR-L3 loop and confirms the observations of the clustering, because four canonical cluster crystal structure medians are located in the same local side-minimum. The other two canonical cluster medians 1F4X and 1G7I are situated in very unfavorable regions of another side-minimum. The most probable macrostates in Figure 8 indicate the existence of various other dominant CDR-L3 loop solution structures.

## Discussion

This present study characterizes the conformational ensemble of the CDR-L3 loop and investigates conformational transitions between different canonical clusters of same length. Structural description of the CDR loops, especially the CDR-H3 and the CDR-L3 loops, are known to be a major challenge for *in silico* development of antibody biotherapeutics because of their diversity in length, sequence and structure (67). Another study focused on characterizing the stability of antigen-binding fragments in dependency of different heavy and light chain pairings and the respective effect on the CDR loop conformational variability. The concept of canonical structures was supported by this investigation, suggesting that the structural repertoire could be diversified by extending beyond the human germline usage (68). The concept of conformational diversity of antibodies and the ability of the same antibody to adopt various conformations was proposed by Pauling and Landsteiner and demonstrated by Milstein and Foote (69–72).

The idea of having ensemble of pre-existing conformations out of which the functional ones are selected was supported by population shift models originating from the Monod-Wyman-Changeux model (73–77). This new view on proteins, i.e., that one sequence can show high structural diversity, facilitated the understanding and evolution of new functions and structures (71). Proper characterization of the CDR loops, especially the loops which are mainly involved in the binding process, is crucial to understand protein-protein interactions and antigen binding. Various studies focused on classifying the CDR loops according to their loop length and sequence composition based on strong experimental structural information (6, 8, 27). We used this experimental support to characterize the CDR-L3 loop ensemble in solution. Four different antibodies with distinct CDR-loop lengths, sequence compositions and types of light chains were used to identify functional solution structures within this ensemble of pre-existing conformations. Figure 1 shows the results of the hierarchical clustering of the first analyzed antibody with the most prominent CDR-L3 loop length of nine residues and displays a high conformational diversity with various transitions between the four observed clusters. Comparison of this result with the six available canonical cluster median crystal structures clearly showed that within one simulated cluster we were able to sample several canonical cluster representatives. Within the highest populated simulated cluster, the assigned canonical cluster representative of L3-9-cis7-1 (cluster median 1J1P) was present. Taking the crystal structure populations into account the L3-9-cis7-1 is the most abundant canonical cluster for all CDR-L3 loop lengths. To compare the populations observed in the PDB with our conformational ensemble in solution we calculated a Markov-state model of the CDR-L3 loop (Figure 2) and found two additional canonical cluster representatives close to the same global minimum of the L3-9-cis7-1 median. The representative of the L3-9-cis7-2 canonical cluster (cluster median 1G7I) is situated in another local side-minimum and displays transition kinetics to the most probable macrostate in the microsecond timescale. Astonishingly, we were also able to sample the transition to the canonical cluster representative of the L3-9-cis7-3 cluster (cluster median 1L7I) in the high micro-to-millisecond timescale. Besides the sampling of conformational transitions between different available canonical clusters we identified an additional macrostate representative which could be an important conformation in solution. The second antibody analyzed has a CDR-L3 loop length of eight residues. Up to now only three canonical clusters could be classified for this length. Again, Figure 3 shows the conformational transitions, as result of the hierarchical clustering, and within the highest populated cluster we identified the assigned canonical cluster L3-8-1 (representative structure 3CMO). With a Markov-state model (Figure 4) we were able to calculate the populations and probabilities of our resulting CDR-L3 loop ensemble and in line with the observations of the first investigated antibody we identified the assigned canonical cluster representative as dominant solution structure. Additionally, we were able to sample transitions between all three canonical clusters in the microsecond timescale. Another potentially important solution structure within this ensemble was identified and is colored gray. The third studied antibody has a CDR-L3 loop length of ten residues and in this case no canonical cluster could be assigned. We compared our hierarchical clustering results (Figure 5) with the three available canonical cluster representatives, which we find within the lowest populated clusters. Besides sampling of available canonical cluster medians, we also identified two highly populated clusters being potentially relevant solution structures. The Markov-state model in Figure 6 reconstructs the kinetics and thermodynamics of the CDR-L3 loop ensemble and identifies a broad and shallow global minimum in which the dominant solution structure is present. The shallow free energy surface observed for this antibody indicates a higher conformational diversity of the CDR-L3 loop most likely originating from the lambda light chain (15). Figure 7 displays the conformational transitions of the last investigated antibody CDR-L3 loop with the length of nine amino acid residues. For this prominent and most common CDR-L3 loop length six canonical clusters were available and compared with our conformational ensemble. Four canonical cluster representatives are sampled within the second highest populated cluster 2 in our simulation. The other two canonical cluster medians were identified in simulated cluster 3. In line with the results in Figure 6, where the canonical cluster representatives are situated in local shallow side-minima, other more probable solution structures dominate in the Markov-state model in Figure 8.

For structure design our results imply, that for a given CDR-L3 loop sequence several canonical structures have to be considered. Our results also indicate that there are dominant CDR-L3 loop structures in solution, that are not apparent from X-ray analysis most likely due to crystal packing effects (31, 32). Further extensive studies of possible solution structures would be needed to decide, whether these dominant structures in solution also can be classified in new canonical structures. It is also evident, that some of the canonical structures indeed belong to the same kinetic minimum in solution (cf. SI Table 1) and thus might be combined.

## Conclusion

We characterized the CDR-L3 loop ensemble in solution for different loop lengths and types of light chains. For four antibodies we were able to structurally, thermodynamically and kinetically profile the conformational space of the CDR-L3 loop in solution. Comparison of the resulting the CDR-L3 loop ensemble with the available canonical structures allowed us to calculate transition kinetics between different canonical clusters. Additionally, we identified all relevant conformations in solution. Our results clearly indicate that the static model of canonical structures should be extended to the description of the CDR-L3 loop as conformational ensemble. These findings have broad implications in the field of antibody structure design, antibody docking and might play a key role in the development of biotherapeutics as they provide a new paradigm in the understanding of CDR-L3 loop conformations and their dynamics.

## Data Availability Statement

All datasets generated for this study are included in the article/Supplementary Material.

## Author Contributions

All authors listed have made a substantial, direct and intellectual contribution to the work, and approved it for publication.

### Conflict of Interest

The authors declare that the research was conducted in the absence of any commercial or financial relationships that could be construed as a potential conflict of interest.

## Abbreviations

CDR: Complementary determining region
Fv: Antibody variable fragment
MD: Molecular dynamics
PCCA: Perron cluster cluster analysis
RMSD: Root mean square deviation
tICA: Time-lagged indpendent component analysis
V_H_: Heavy chain
V_L_: Light chain.
