# Supplementary material for: Transitions of CDR-L3 Loop Canonical Cluster Conformations on the Micro-to-Millisecond Timescale
Source: Front Immunol. 2019 Nov 19;10:2652. doi: 10.3389/fimmu.2019.02652 (PMC6877499; doi:10.3389/fimmu.2019.02652)
Supplement: Supplementary file 1 [file Table_1.DOCX]

Table 1: Classification of kinetic minima in solution for eight antibody fragments with CDR-L3 loop length of 9 into canonical structures and non-canonical solution structures (NCSS). The most dominant kinetic minimum in solution is denominated as C1 for each antibody. Less populated kinetic minima in solution are named C2, C3 and C4 for each antibody individually. Populations of kinetic minima in solution are mentioned in brackets and indicated by different shades of grey. The same kinetic minimum reoccurring for several canonical structures indicates that these canonical structures belong to the same kinetic minimum in solution. This, e.g., suggests that L3-9-2 and L3-9-cis7-1, which are the highest populated canonical structures in the PDB with CDR-L3 loop length of 9, actually should be seen as one kinetic minimum in solution, as they coincide for all of the eight sequences studied.

| CDR-L3 9 | L3-9-1 (1F4X) | L3-9-2 (1KCS) | L3-9-cis6-1 (2FBJ) | L3-9-cis7-1 (1J1P) | L3-9-cis7-2 (1G7I) | L3-9-cis7-3 (1L7I) |  | NCSS |
| --- | --- | --- | --- | --- | --- | --- | --- | --- |
| 3EOA | 0% | C1 (89%) | 0% | C1 (89%) | C1 (89%) | C2 (11%) |  | 0% |
| 3L5W | C2 (14%) | C1 (77%) | 0% | C1 (77%) | C2 (14%) | C4 (3%) |  | C3 (6%) |
| 1MLB | C3 (14%) | C2 (23%) | C3 (14%) | C2 (23%) | C3 (14%) | C3 (14%) |  | C1+C4 (63%) |
| 1FL6 | C2 (18%) | C2 (18%) | C2 (18%) | C2 (18%) | C2 (18%) | C1 (76%) |  | C3 (6%) |
| 1HIM | C4 (4%) | C3 (7%) | C3 (7%) | C3 (7%) | C4 (4%) | C3 (7%) |  | C1+C2 (89%) |
| 3RVW | 0% | C1 (61%) | C1 (61%) | C1 (61%) | C2 (32%) | C4 (2%) |  | C3 (5%) |
| 1NGZ | C2 (22%) | C2 (22%) | 0% | C2 (22%) | C2 (22%) | 0% |  | C1+C3 (78%) |
| 1AJ7 | 0% | C1 (54%) | C2 (18%) | C1 (54%) | C1 (54%) | C1 (54%) |  | C3+C4 (28%) |
